# Supplementary material for: Functional Characterization of Aldehyde Dehydrogenase in Fusarium graminearum
Source: Microorganisms. 2023 Nov 28;11(12):2875. doi: 10.3390/microorganisms11122875 (PMC10745421; doi:10.3390/microorganisms11122875)
Supplement: Supplementary file 1 [file microorganisms-11-02875-s001.zip › microorganisms-2682965-supplementary.pdf]

Table S1. The primer sequences used in this study.

Primer Name	Sequence (5′—3′)	Amplification Length	Annealing Temperature
QTublinF	GTCAGTGCGGTAACCAAATCGGT	95 bp	60 °C
QTublinR	CTCAGAGGTGCCGTTGTAAACACC		
Q00217F	GACCGCACCTTTCCCTGTT	264 bp	60 °C
Q00217R	AGCCTCGGACCTATCCTGTG		
Q03873F	ATGAGCAGGTGGTTAGAAGTGG	83 bp	60 °C
Q03873R	TGGCTGTAAAGTCAATCGTGATG		
Q03649F	CCAAAGGCTATTCGGTTATGC	294 bp	60 °C
Q03649R	TGAGAACTGCTTGTCGATGTATG		
Q02322F	GTCGGTGGCGGTCTTATCA	155 bp	60 °C
Q02322R	CCCGTAGGCACATCACTTTG		
Q09595F	CGTGCTCAGCCTTGTGGTT	237 bp	60 °C
Q09595R	TTGGGAAGTGTTGCCTGAATAG		
Q02263F	TACTTCTGCCTGGGGCTACG	114 bp	60 °C
Q02263R	GTGACAACGCCTCCCTCTTT		
Q03703F	TTTACCAGATTTACCCTTCGTCC	216 bp	60 °C
Q03703R	GTTCAACATCAGCCATAGTTCCAT		
K04194 1F	CCCAGAAGCCACAACATC	619 bp	49 °C
K04194 2R	CTTGCCTCACCAACCCT		
K04194 3F	TGGCGATGATAGCAAGA	314 bp	45 °C
K04194 4R	AACAATGAGGAAGGACGA		
K04194 5F	ATGTCAGACTTGTACGTTAACC	1610 bp	57 °C
K04194 6R	TCACAGACGAGAACCCAAGTT		
K04194 7F	GGCTTGCCTTGCTCCAC	768 bp	51 °C
H855 R	GCTGATCTGACCAGTTGC		
H856 F	GTCGATGCGACGCAATCGT	562 bp	51 °C
K04194 8R	ATAGTCGGGTGGTGGAAG		
K00139 1F	GCCAAGATTTGAGGGATAA	238 bp	49 °C
K00139 2R	TGACGGACAAGATAGGGA		
K00139 3F	TTTGATGGTGGTGATGGG	352 bp	49 °C
K00139 4R	TCTGTTACCGAGATTGCTG		
K00139 5F	ATGTCTCCCCCCTCAATC	1560 bp	51 °C
K00139 6R	CTACAGCCTATTTCCCATAT		
K00139 7F	CCAGCGAGACAGGATTAGA	424 bp	51 °C
H855 R	GCTGATCTGACCAGTTGC		
H856 F	GTCGATGCGACGCAATCGT	977 bp	55 °C
K00139 8R	GCAACCCTCCCATCCTCAA		
K11482 1F	CCACTACCACCACCAACA	413 bp	49 °C
K11482 2R	CTCGGAGCCAAGATTTATT		
K11482 3F	GATGAATCTAAGGTCCAGTCC	600 bp	53 °C
K11482 4R	CCGTCTTTGTGCCGATC		
K11482 5F	ATGGCGCATACGGATGTG	1462 bp	51 °C

K11482 6R	CTACCCATACAGCTGGGA		
K11482 7F	CCCACTACCACCACCAA	947 bp	51 °C
H855 R	GCTGATCTGACCAGTTGC		
H856 F	GTCGATGCGACGCAATCGT	1885 bp	55 °C
K11482 8R	TCTCAACATGCGGAAGC		
K05375 1F	CATCTTCACCAGGCATAA	1482 bp	45 °C
K05375 2R	CCGTCAAGACCCGTTA		
K05375 3F	GATTTCGTGGTCGGTCTA	145 bp	49 °C
K05375 4R	TTCCGTCAAACTCTTCTCAT		
K05375 5F	ATGACGTTCCCATCCGATAT	1530 bp	55 °C
K05375 6R	CTACAATTTGGAGAAAACACCC		
K05375 7F	AGCAGGCAGGCAAGGA	1767 bp	47 °C
H855 R	GCTGATCTGACCAGTTGC		
H856 F	GTCGATGCGACGCAATCGT	681 bp	45 °C
K05375 8R	TTGGGTCGCCTTTAT		
K00979 1F	GGTGGGTTTGGCTGTGG	454 bp	54 °C
K00979 2R	GTAACTGTCTCGGCTTCTGG		
K00979 3F	GTTTGCCAATCAATCCAC	990 bp	47 °C
K00979 4R	TGTTTCGCCCTCGTTAG		
K00979 5F	ATGGCTCTCACCGTCGAGCTGT	1594 bp	52 °C
K00979 6R	TTAGTACAGCTTGATGGC		
K00979 7F	TTAGCAAATAACACCGACTG	1137 bp	51 °C
H855 R	GCTGATCTGACCAGTTGC		
H856 F	GTCGATGCGACGCAATCGT	1314 bp	55 °C
K00979 8R	GTATGTATTAGGTGAGGGAGG		
K02160 1F	CGTTGGATGTTGGGACTG	467 bp	51 °C
K02160 2R	GGGAACCTGGAGGAAAGC		
K02160 3F	ATTGGGAATGACTTGGCTAT	311 bp	51 °C
K02160 4R	CGTTATCTGGAGATGTGGG		
K02160 5F	ATGACATCAACTACACAAATC	1701 bp	50 °C
K02160 6R	CTATAATTCAAATCCCAAGTT		
K02160 7F	TGCGAAACTCCTCCACCA	921 bp	51 °C
H855 R	GCTGATCTGACCAGTTGC		
H856 F	GTCGATGCGACGCAATCGT	1043 bp	55 °C
K02160 8R	AACAGTGAGGTCGTAGTAGCC		
K05831 1F	TGAGACTGATAGAGGAGGACC	794 bp	50 °C
K05831 2R	CTTGGCACGCATAACG		
K05831 3F	AACAGCGAACTCGGTCA	702 bp	47 °C
K05831 4R	TCAAAGCCCTTCATCCC		
K05831 5F	ATGGCAGAAATTACCATCAC	1591 bp	49 °C
K05831 6R	TCACAAGTTGAAAACATTAAT		
K05831 7F	AAAGCCACAGCCATCTC	1745 bp	47 °C
H855 R	GCTGATCTGACCAGTTGC		
H856 F	GTCGATGCGACGCAATCGT	2081 bp	52 °C

K05831 8R	TCTGCTGTCGGAAACCC		
HYG/F	GGCTTGGCTGGAGCTAGTGGAGGTCAA	765 bp	45 °C
HY/R	GTATTGACCGATTCCTTGCGGTCCGAA		
YG/F	GATGTAGGAGGGCGTGGATATGTCCT	921 bp	53 °C
HYG/R	AACCCGCGGTCGGCATCTACTCTATTC		
C04194 F	CAGCCACCTTTCCCCACGTCT	2614 bp	59 °C
C04194 R	CAGACGAGAACCCAAGTTGAC		
GFP-Hind3-F	ATGGTGAGCAAGGGCGAG	758 bp	53 °C
GFP-BamHI-R	TTACTTGTACAGCTCGTCCA		
